# Flip-Flop of Phospholipids in Proteoliposomes Reconstituted from Detergent Extract of Chloroplast Membranes: Kinetics and Phospholipid Specificity

**DOI:** 10.1371/journal.pone.0028401

**Published:** 2011-12-12

**Authors:** Archita Rajasekharan, Sathyanarayana N. Gummadi

**Affiliations:** Department of Biotechnology, Indian Institute of Technology Madras, Chennai, India; Institute of Molecular and Cell Biology, Singapore

## Abstract

Eukaryotic cells are compartmentalized into distinct sub-cellular organelles by lipid bilayers, which are known to be involved in numerous cellular processes. The wide repertoire of lipids, synthesized in the biogenic membranes like the endoplasmic reticulum and bacterial cytoplasmic membranes are initially localized in the cytosolic leaflet and some of these lipids have to be translocated to the exoplasmic leaflet for membrane biogenesis and uniform growth. It is known that phospholipid (PL) translocation in biogenic membranes is mediated by specific membrane proteins which occur in a rapid, bi-directional fashion without metabolic energy requirement and with no specificity to PL head group. A recent study reported the existence of biogenic membrane flippases in plants and that the mechanism of plant membrane biogenesis was similar to that found in animals. In this study, we demonstrate for the first time ATP independent and ATP dependent flippase activity in chloroplast membranes of plants. For this, we generated proteoliposomes from Triton X-100 extract of intact chloroplast, envelope membrane and thylakoid isolated from spinach leaves and assayed for flippase activity using fluorescent labeled phospholipids. Half-life time of flipping was found to be 6±1 min. We also show that: (a) intact chloroplast and envelope membrane reconstituted proteoliposomes can flip fluorescent labeled analogs of phosphatidylcholine in ATP independent manner, (b) envelope membrane and thylakoid reconstituted proteoliposomes can flip phosphatidylglycerol in ATP dependent manner, (c) Biogenic membrane ATP independent PC flipping activity is protein mediated and (d) the kinetics of PC translocation gets affected differently upon treatment with protease and protein modifying reagents.

## Introduction

Biogenic membranes (self-synthesizing) such as endoplasmic reticulum (ER) and Golgi complex of animal cells and yeasts are capable of synthesizing their own phospholipids (PLs). Most of the PL synthesizing enzymes are localized in the cytosol and the newly synthesized PLs are initially localized on the cytoplasmic side of the ER [1]–[3]. Some of the newly synthesized PLs need to flip to other side (lumen) for membrane biogenesis and propagation [4], [5]. The flip-flop movement of PLs is a thermodynamically unfavorable process because of the movement of charged head groups of PLs across the hydrophobic lipid bilayer [6]. Nevertheless lipid translocation is essential during physiological processes such as apoptosis, blood coagulation etc. This process requires metabolic energy usually derived from ATP hydrolysis [7]. The half-life time of PL translocation in artificial membranes or liposomes varies between few hours to days [6]. It has been experimentally shown that PL flip-flop in biogenic membranes occur rapidly (half-time∼few minutes) [8]–[13]. These studies also reveal that PL translocation in biogenic membrane occurs bi-directionally independent of PL head group by a facilitated diffusion process without the requirement of metabolic energy input, and in the presence of specific membrane proteins [11]–[13]. ER of animals, yeasts and bacterial cytoplasmic membranes (bCM) of *Eschericia coli* and *Bacillus subtilis* are equipped with special PL translocators [12], [14], [15]. These translocators have been termed as biogenic membrane flippases and they differ from metabolic energy dependent transporters (ATP binding cassette transporters and multidrug resistant proteins), which are localized in non-biogenic membranes such as plasma membrane (PM). These ATP dependent PL translocators are of two types, viz., flippases and floppases. Flippases are involved in vectorial transport of aminophospholipids from exoplasmic leaflet to cytosolic leaflet (e.g. aminophospholipid translocase) [16], [17]. Floppases are involved in vectorial transport of phosphatidylcholine (PC) and other choline containing lipids from cytosolic to exoplasmic leaflet (multidrug resistant proteins) [16]. PM is also equipped with scramblases which get activated by elevated levels of calcium and function in an ATP independent manner scrambling PLs across the bilayer membrane [18], [19]. Most of the PM lipid translocators have been identified and characterized. However, no biogenic membrane flippases have been identified so far.

The membrane PL synthesis and their distribution to various organelles are well understood in animals and yeast. However, this process is poorly understood in plants. In plants, the synthesis of membrane PLs is more complex than bacteria and animals and occurs in ER and chloroplasts [20]. The fatty acyl groups are synthesized by enzymes present in chloroplast and after being converted to acyl-CoA, move to ER, where it is utilized in the synthesis of all membrane PLs like PC, phosphatidylethanolamine (PE), phosphatidylserine (PS) and free sterols [20], [21]. However, phosphatidylglycerol (PG) is synthesized and localized in the chloroplast. The bulk of PLs and sterols synthesized in ER move to other organelles for membrane biogenesis and also provide chloroplasts with acyl-glycerol back bone for the synthesis of the bulk of thylakoid lipids [21], [22]. In contrast to animal cells, plant cell membranes have distinct non-phosphorus glycoglycerolipids such as monogalactosyl diacylglycerol (MGDG), digalactosyl diacylglycerol (DGDG) and sulfoquinovosyl diacylglycerol (SQDG) in chloroplast membranes. These membrane lipids are synthesized on the inner and outer envelope of chloroplast membranes from precursors present in the plastids or trafficked to the plastids from ER [23], [24]. Due to involvement of different sub-cellular and endo-membrane system in biosynthesis of lipids, there is a constant shuttling of precursors between biogenic organelles which adds to its complexity. Till date, no detailed studies are available on translocation of PLs in various cellular membranes and their kinetics and substrate specificity. In an earlier study, we showed first evidence for biogenic membrane flippase activity in ER of spinach leaves [25]. However, till date there is no evidence on translocation of PLs in chloroplast envelope membranes.

In this study intact chloroplast, envelope membranes and thylakoids from spinach leaves were isolated and the detergent extract of these membranes were reconstituted into proteoliposomes and studied for biogenic membrane flippase (ATP independent), ATP dependent flippase and scramblase activity. In addition we also studied the kinetics of PL translocation and PL specificity for the various flippase activities. Our results clearly showed the presence of both, ATP independent and ATP dependent pool of flippases and that the PC flippase activity was protein-mediated and sensitive to NEM a cysteine modifier. We also confirmed that the translocation of lipids was not due to the presence of Ca^2+^ dependent scramblases.

## Materials and Methods

### Materials

Egg phosphatidylcholine (ePC), sodium dithionite, calcein, diethyl pyrocarbonate (DEPC), bovine pancreatic trypsin, phenyl glyoxal, 4-(2-aminoethyl)benzenesulfonyl fluoride (AEBSF), N-Ethylmaleimide (NEM), Guanosine 5′-triphosphate (GTP) sodium salt, ULTROL-grade Triton X-100, the protein estimation kit (BCA kit), Percoll, Plant Protease Inhibitor Cocktail were obtained from Sigma. 1-Oleoyl-2-{12-(7-nitro-2-1,3-benzoxadiazol-4-yl)aminododecanoyl}-*sn*-glycero-3-phosphocholine (NBD-PC), 1-oleoyl-2-{6-[(7-nitro-2-1,3-benzoxadiazol-4-yl)amino]hexanoyl}-sn-glycero-3-[phospho-rac-(1-glycerol)] ammonium salt (NBD-PG) were purchased from Avanti Polar Lipids, SM-2 Biobeads was purchased from Bio-Rad Laboratories. Adenosine 5′-triphosphate (ATP) disodium salt was purchased from Biobasic. All other chemicals were procured from Himedia, India.

### Isolation of chloroplast membranes from *Spinacia oleracea* leaves

Intact chloroplasts were isolated from spinach leaf homogenate by multiple percoll gradient protocol as described earlier [26]. Briefly, 30 g spinach leaves were homogenized in organelle isolation buffer (1% (w/v) polyvinylpolypyrrolidone, 300 mM D-sorbitol, 50 mM HEPES, 2 mM Na-EDTA, 1 mM MgCl_2_ and 0.1% BSA in the presence of a 0.1% v/v plant protease inhibitor) in a blender with two 30 s bursts. The homogenate was filtered through 8 layers of cheese cloth. Chloroplasts were sedimented by centrifugation at 1500×*g* in a swinging bucket rotor. The crude chloroplast pellet was resuspended in 3 ml resuspension buffer containing 300 mM D-sorbitol, 50 mM HEPES, 2 mM Na-EDTA, 1 mM MgCl_2_ and 0.1% BSA. The resuspended sample was loaded onto a multiple percoll density gradient (4 ml 10% Percoll, 4 ml 40% Percoll, 4 ml 80% Percoll from top to bottom) prepared in resuspension buffer. The Percoll gradients were centrifuged for 20 min at 16,000×*g* in SW 41Ti rotor. Broken chloroplasts were collected at the 10–40% interface, while intact chloroplasts formed a band at the 40–80% interface. The fractions containing intact chloroplasts were collected and combined using a thin pasteur pipette and washed twice with three volumes of washing buffer (300 mM D-sorbitol, 50 mM HEPES, 2 mM Na-EDTA, 1 mM MgCl_2_) and centrifuged for 10 min at 1,500×*g* and aliquots were snap frozen and stored at −80°C. All steps were carried out at 4°C and fractions were collected at every step.

Intact chloroplasts were fractionated into envelope and thylakoid membranes as described earlier [27]. Purified intact chloroplasts (∼30 mg chlorophyll) were lysed by incubation with 20 ml of 10 mM Tricine/NaOH pH 7.5 containing 4 mM MgCl_2_ by 2–3 freeze thaw cycles. The broken chloroplasts were adjusted to 0.3 M sucrose and layered onto a discontinuous sucrose gradient made of 12 ml 0.98 M sucrose and 16 ml of 0.6 M sucrose buffered with 10 mM Tricine/NaOH pH 7.5 containing 4 mM MgCl_2_. The gradient was centrifuged at 113000×g_max_ for 90 min at 4°C in a Beckman SW 27 rotor. The stromal proteins remain at the top of the gradient while the envelope membranes form a band at the 0.6/0.98 M sucrose interface and the thylakoid membranes form a pellet at the bottom of the gradient. Envelope membranes were recovered by dilution followed by centrifugation at 90000×*g*_max_ for 1 h in Beckman 100 Ti rotor. The collected membranes were buffered to 0.3 M sucrose, washed twice and resuspended in 10 mM Tricine/NaOH pH 7.5 containing 4 mM MgCl_2_ and stored at −80°C.

### Purity of isolated intact chloroplasts

#### Chlorophyll estimation

Aliquots of the isolated fractions were extracted with 80% acetone and optical density readings were recorded at 652 nm, 664.5 nm and 647 nm in a JASCO spectrophotometer. Chlorophyll concentrations were calculated as described earlier [28]. Calculations were done by two methods, in the first method total chlorophyll was measured and in the second method chlorophyll a and chlorophyll b was separately calculated using the following formulae
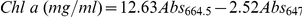


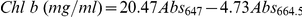


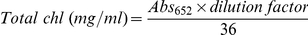



#### Mg^2+^ dependent ATPase assay (chloroplast marker)

ATPase activity was measured as the amount of P_i_ released by ATP hydrolysis using Ames method [29]. Briefly 50 µg of protein was taken and final volume was made to 100 µl with water and added to 200 µl of reaction mixture containing 3 mM ATP, 3 mM MgSO_4_.7H_2_O, 50 mM KCl, 1 mM sodium molybdate and 0.02% Brig 58, buffered to pH 8.0 with 50 mM Tricine. In addition, 3 mM sodium azide and 0.25 mM sodium orthovanadate were added to inhibit mitochondrial F-type ATPase and PM P-type ATPase respectively. The mixture was incubated at 37°C for 20 min. The reaction was stopped by adding 50 µl of 25% ice-cold TCA and 300 µl of 1% (w/v) SDS with samples on ice bucket. The white precipitates formed were removed by centrifuging at 10,000×*g* for 10 min. The supernatant was transferred to fresh tubes to estimate P_i_ released by adding 1 ml of Ames reagent (6 parts of 0.4% w/v ammonium molybdate in 0.5 M H_2_SO_4_ to 1 part of 10% w/v ascorbic acid) and incubated for 30 min at room temperature. The absorbance at 820 nm was measured against reagent blank.

#### Cytochrome c reductase assay (ER marker)

Different sub-cellular fractions collected during isolation were analyzed for cytochrome *c* reductase (CCR) activity, the marker enzyme specific to ER. Briefly, 50 µL of the purified fraction was added to 700 µl of assay mixture containing 300 mM Tris buffer (pH 8.5), 20 mM potassium bicarbonate buffer (pH 8.5), 0.5 mg/mL BSA, 1% (w/v) cytochrome *c* solution, and 7.05 mM NADH. The reduction of cytochrome *c* was monitored spectrophotometrically by measuring the increase in absorbance at 550 nm for 10 min in a JASCO spectrophotometer [25].

#### P-type ATPase assay (plasma membrane marker)

The ATPase activity was measured in 50 mM MES, 10 mM MgS0_4_.7H_2_O, 5 mM sodium azide, 0.2 mM ammonium molybdate, 100 mM KNO_3_ and 3 mM ATP in the presence and absence of 100 µM sodium orthovanadate which is a PM ATPase inhibitor. ATPase activity was measured as the amount of Pi released by ATP hydrolysis as described in the previous section.

### Microscopic study

The isolated chloroplast fraction was also analyzed using a scanning electron microscope (SEM) for intactness. The sample was fixed, air dried and coated with gold particles using an ion sputter coating unit. The EDS detector system was used and samples were slowly cooled under vacuum on the peltier stage after which it was observed in a Hitachi S-3400N model SEM [30].

### Preparation of Triton X-100 Extract (TE) from fractionated chloroplast membranes

TE was prepared by adding 250 µl of fractionated chloroplast membrane with equal volume of 2× reconstitution buffer (10 mM HEPES/NaOH pH 7.5, 100 mM NaCl and 1% (w/v) Triton X-100) respectively. The sample was mixed and kept at 4°C with end over end rocking for 45 min. It was then ultra centrifuged in a MLA 130 rotor at 1,75,000×*g* for 30 min at 4°C. The supernatant was carefully removed and used directly for reconstitution at a protein concentration of ∼5 mg/ml.

### Reconstitution of liposomes and proteoliposomes

Liposomes and proteoliposomes were prepared as described previously [13], [25]. 4.5 µmol ePC and 0.3 mol% of NBD-PC were dried under a stream of nitrogen and solubilized in reconstitution buffer. For preparation of proteoliposomes, TE (∼5–6 mg/ml) was added to solubilized lipid samples with known protein concentration. Pretreated SM2 biobeads were used to remove Triton X-100 to form liposomes or proteoliposomes. The reconstituted liposomes and proteoliposomes were collected by ultracentrifugation in MLA 130 rotor at 2,30,000×*g* for 45 min at 4°C and washed 3 to 4 times with assay buffer (10 mM HEPES/NaOH pH 7.5, 100 mM NaCl) to remove background fluorescence due to non-reconstituted lipids and proteins. The vesicles were resuspended in 1 ml of same buffer and passed 11 times through a 0.1 µm polycarbonate membrane filter using a lipid extruder to make vesicles of uniform size. The protein/phospholipid ratio (PPR) of proteoliposomes was determined as previously described [31], [32].

### Vesicle size analysis by dynamic light scattering (DLS)

The particle size (average mean diameter) of liposomes and proteoliposomes was measured by dynamic light scattering (DLS) at an angle of 90°C at 25°C for 5 min in a 90Plus particle size analyzer, Brookhaven Instruments Corp., New York, USA. Diluted liposome and proteoliposome samples (2.5 ml) were analyzed before and after extruding through a 0.1 µm membrane filter [33].

### Sucrose floatation gradient analysis

This analysis was performed to verify the reconstitution of membrane proteins from TE as described previously [25], [34]. Briefly, 1 ml of the reconstituted proteoliposomes was layered under a series of sucrose (w/w) [20, 10, 5, 2.5%] and centrifuged at 100000×*g* in a swinging – out rotor (SW 4Ti) for 1 h. Fractions were collected from different sucrose interfaces and dialyzed against assay buffer for 24 h with 4–5 buffer changes to remove sucrose from the fractions. The fractions were analyzed for the estimation of protein and phospholipids as described previously [31], [32].

### Collisional quenching experiments

To confirm the steady-state distribution of the labeled PLs across the membranes of liposomes and proteoliposomes, collisional quenching of NBD probes with potassium iodide (KI) was performed according to the method previously described [35]. NBD-PC containing liposomes and proteoliposomes were reconstituted using reconstitution buffer supplemented with 0.3 M KCl. Excitation and emission wavelengths were set at 470 and 530 nm, respectively. Fluorescence intensity was measured for samples consisting of 50 µl of vesicles diluted into 1.95 ml of buffer containing 10 mM HEPES-NaOH (pH 7.5), and the quencher KI (0 to 0.3 M). Data were analyzed according to the modified Stern -Volmer equation: 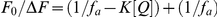 where, *F*_o_ is the fluorescence intensity in the absence of the quencher, Δ*F* is the fluorescence intensity in the presence of the quencher at concentration [*Q*], *f_a_* is the fraction of fluorescence which is accessible to the quencher, and *K* is the Stern-Volmer quenching constant [36]. The fraction of NBD-PC accessible to iodide quenching is calculated as the inverse of the Y - intercept.

### Flippase assay

The assay was performed using Perkin Elmer LS–55 Fluorescence spectrophotometer as described earlier [25]. A total of 20 µl of NBD-PC labeled liposomes and proteoliposomes was added to 1.98 ml of assay buffer in a fluorescence cuvette. Time-dependent fluorescence (Excitation at 470 nm and Emission at 530 nm) was monitored with constant low-speed stirring at 22°C. After the stabilization of fluorescence intensity (∼100 s), sodium dithionite (1 M prepared freshly in Tris base pH 10) was added to a final concentration of 2 mM and decrease in fluorescence was measured for ∼400 s. The initial fluorescence (F_0_) was taken as the average value of fluorescence intensity of the first plateau; the fluorescence after dithionite reduction (F_t_) was taken as the average value of the second plateau. The % of NBD-PC reduced (P_red_) upon dithionite addition was calculated by normalizing the *F*_0_ to 1.0 and *F_t_* as the fraction of *F*_0_. The activity of flippase in a proteoliposome preparation was taken as the difference between the percent reduced in the sample and the percent reduced in a liposome sample (control). Briefly,
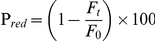


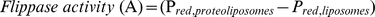



### Assay for ATP dependent flippase activity

Liposomes and proteoliposomes were prepared as above with 4.5 µmol ePC and 0.3 mol% of NBD-PG and inside labeled vesicles were prepared by treating with 20 mM dithionite (Figure S1) and washing twice with assay buffer as described above. The vesicles were assayed for flippase activity as described earlier after incubating at 37°C with freshly prepared ATP (5 mM final concentration). The inside labeled vesicles were also subjected to protein modification in presence of ATP as described below.

### Treatment of TE and proteoliposomes with protease

Freshly prepared stock solution (50 mg/ml) of bovine pancreatic trypsin was added to TE to yield a final concentration of 10 mg/ml and incubated at 37°C for 30 min before reconstitution. Similarly, the intact vesicles were treated with trypsin as described previously [12], [25]. Briefly, 50 µL of liposomes (control) or proteoliposomes was added with desired final concentrations of trypsin in assay buffer in assay mixture of 200 µL and incubated at 37°C for 30 min. The incubated sample was then added to 2 mL of assay buffer, and the fluorescence intensity was monitored as described previously.

### Treatment of TE and proteoliposomes with protein modification reagents

Freshly prepared stock solutions (typically 100 mM) of diethyl pyrocarbonate (DEPC) in water, N-Ethylmaleimide (NEM), phenyl glyoxal (PG), and 4-(2-aminoethyl)benzenesulfonyl fluoride (AEBSF) in assay buffer were added to the liposomes (control) or proteoliposomes to yield the desired final concentration of protein modification reagent. The modifiers were first titrated with a known amount of proteoliposomes to determine the effective working concentrations of these reagents. Then the proteoliposomes with a known quantity of protein were used to study the effect of these compounds on flippase activity. TE was also treated with highest concentration of protein modifier, 20 mM of DEPC, 40 mM NEM, 5 mM of AEBSF and 5 mM of PG respectively. In all experiments, vesicle integrity was determined by treating the control liposomes with the largest amount of reagent used in the experiment and monitoring the fluorescence intensity for 400 s after dithionite treatment followed by treatment with Triton X-100. The proportion of functional flippases eliminated by treatment with protein modifying reagents (% inhibition) was calculated from the percent change in *P*_red_ after subtracting *P*_red_ for liposomes [13]: 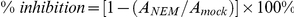.

### Assay for scramblase activity

The measurement of scramblase activity was performed by two methods as described previously [37] with slight modification. Briefly, in the first method symmetrically labeled proteoliposomes were prepared as described in the previous section. Then these vesicles were treated with a final concentration of 20 mM dithionite for 5 min to quench the fluorescent lipid molecules of the outer leaflet and immediately centrifuged for 20 min at 2,30,000×*g* to pellet down the inside-labeled vesicles. Proteoliposomes containing inside labeled NBD-PC was incubated for 2 h at 37°C in assay buffer in presence of 2 mM Ca^2+^ or 4 mM EGTA (control) and then transferred to a stirred fluorescence cuvette thermostatted at 22°C. The initial fluorescence was recorded for 200 s, and 2 mM dithionite (final concentration) was added. The difference between the non-quenchable fluorescence observed in the presence and absence of Ca^2+^ contributed to the Ca^2+^-induced scrambling of NBD-PC located in the inner leaflet of proteoliposomes. In the second method the proteoliposomes were formed using only ePC without any fluorescent lipid. Outside labeled vesicles were prepared by adding NBD-PC (final solvent concentration of 0.25% DMSO) to pre-formed proteoliposomes. It was then incubated for two minutes at 37°C and vesicles were pelleted at 2,30,000×*g* and washed twice with assay buffer. The vesicles were then incubated with 2 mM Ca^2+^ or 4 mM EGTA and assay was performed as described above at time (t) = 0, 1, 2 and 3 hours of incubation.

### Statistical Analysis

Data are presented as the mean of at least three independent experiments along with standard deviation (SD). Statistical analysis of data was done by Student's *t* test using Stata 10. Probability values (*p*) of <0.05 were considered to be statistically significant.

## Results

### Flippase assay using fluorescent PL analogs

**Figure 1:**
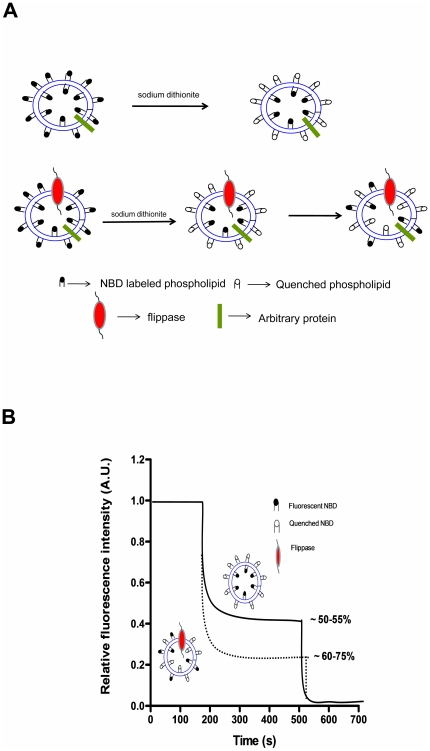
Schematic representation of flippase assay. Flippase activity is measured by quenching fluorescently labeled PLs by sodium dithionite. (A) Energy independent activity -Reconstituted liposomes and proteoliposomes without flippase when treated with sodium dithionite, quenches half ∼50–55% the NBD-labeled PLs. In vesicles reconstituted with a functional flippase capable of bi-directional PL translocation there is a second phase of slow quenching of NBD labeled lipids to >55–100% (B) On addition of 0.1% (w/v) Triton X-100 to the symmetrically labeled liposomes (straight line) and proteoliposomes (dotted line) the vesicles are disrupted making all the PLs accessible to dithionite showing ∼100% quenching.

The flippase assay is based upon the ability of dithionite to reduce the NBD labeled PL fluorophore to non-fluorescent amine products and measurement of flippase activity depends upon amount of fluorescent PLs available for dithionite quenching on the outer leaflet (Figure 1A). In the case of liposomes or proteoliposomes which do not have flippase but reconstituted with arbitrary protein, all the PLs on the outer leaflet were quenched upon addition of dithionite and yielded ∼52±3% quenching, whereas inner leaflet PLs were protected from dithionite (Figure 1A). Time dependent fluorescence quenching can be fitted to single exponential decay kinetics as reported earlier [12], [25]. When proteoliposomes containing NBD-PC were reconstituted with detergent solubilized membrane fractions containing flippase activity, two-phase exponential decay kinetics was observed (Figure 1B). A sharp decrease in NBD fluorescence to 50% followed by a slow decrease in fluorescence was observed. This suggests that the sharp decrease in fluorescence is due to quenching of labeled lipids on outer leaflet (Fig. 1B trace a), whereas the slower decrease in fluorescence is due to inner labeled PLs being flipped to the outer leaflet which subsequently gets quenched by dithionite (Figure 1B trace b, dotted line). However, upon addition of 0.1% (w/v) Triton X-100 to liposomes and proteoliposomes, ∼100% quenching was observed, indicating that the membrane permeability was disturbed leaving all the PLs accessible to dithionite for quenching (Figure 1B).

### Isolation of spinach chloroplast membranes

**Figure 2:**
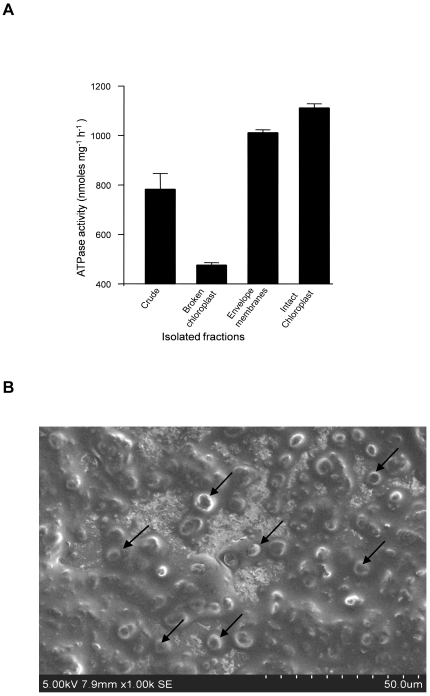
Isolation of intact spinach chloroplast membrane. (A) ATPase activity in isolated fractions. ATPase activity was measured as the amount of P_i_ released by ATP hydrolysis using Ames reagent which is measured at 820 nm. Mean values and the range from duplicate samples within one representative experiment are presented (B) SEM image of intact chloroplast (represented by arrows) which were fixed and coated with gold particles was observed under1000× magnification.

Multiple percoll gradients were used to isolate intact chloroplast from spinach leaves as described previously. Thylakoids and envelope membranes were isolated by lysing the intact chloroplast by freeze thaw in buffer and fractionated on a sucrose gradient as mentioned above. Several researchers have used both chlorophyll content and Mg^2+^ ATPase as marker for chloroplast isolation [28], [38], [39]. Hence, in this study the purity of isolated chloroplast membranes were confirmed by performing these specific marker assays. In order to check if the chloroplast membrane fractions isolated were contaminated with other organelles we checked the cytochrome c reductase (CCR) and P-type ATPase assay in presence and absence of vanadate which are specific for ER and PM respectively. The results clearly showed that chlorophyll content (chlorophyll a, chlorophyll b and total chlorophyll) and chloroplast ATPase activity was enriched in the intact chloroplast fraction when compared to crude by ∼27 times (data not shown) and ∼1.3 fold increase respectively (Figure 2A). Also thylakoids showed high levels (∼25 times compared to crude, data not shown) of chlorophyll (thylakoid marker) and envelope membranes showed ATPase activity comparable to the amount observed in intact chloroplast. The activity of CCR and P-type ATPase was negligible in the crude and not detected in the final chloroplast fraction (data not shown). SEM analysis was performed which revealed that the chloroplast fraction was pure and intact (Figure 2B). These results suggest that the membrane isolated fractions were highly pure with negligible contamination.

### Reconstitution of spinach chloroplast membranes into proteoliposomes

**Figure 3:**
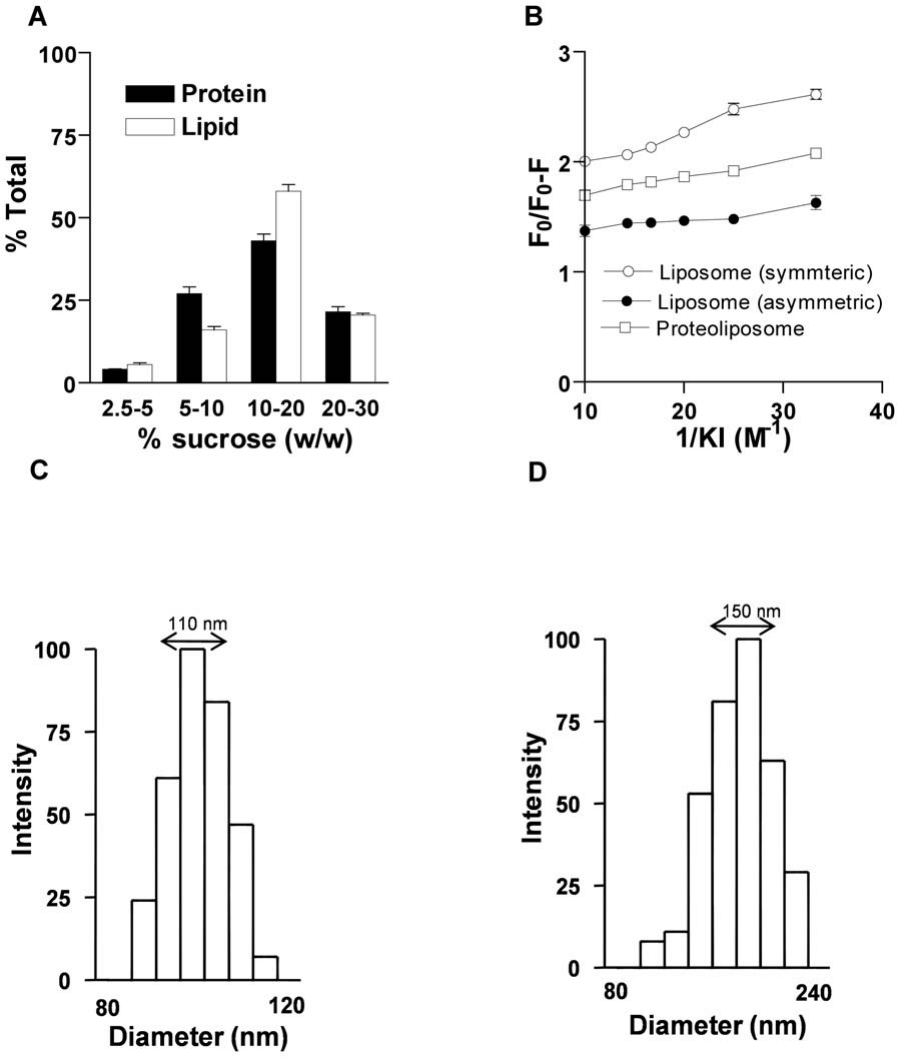
Confirmation of vesicle intactness. (A) Sucrose floatation gradient analysis, x-axis shows the interfaces between two sucrose step gradients and y-axis shows the corresponding percent total lipid and protein at each interface. (B) Collisional quenching of NBD fluorescence with iodide ions to determine the fraction of NBD-PC accessible on the outer leaflets of vesicles. Proteoliposomes (open squares) and liposomes (open circles) were reconstituted from Triton X-100-solubilized mixtures containing NBD-PC. Asymmetrically labeled liposomes (filled circles) were prepared by adding NBD-PC to preformed vesicles. The data are presented as modified Stern-Volmer plots, *F*_o_ is the fluorescence intensity of the sample in the absence of quencher, whereas Δ*F* is the fluorescence intensity at a given iodide ion concentration. The inverse of the *y*-intercept represents the fraction of NBD-PC that is accessible to the quencher. The assay was performed 2 independent times and p calculated was <0.01 (C&D) Liposomes and proteoliposomes were analyzed in 90Plus particle size analyzer. The DLS measurements showed liposomes (C) to be 110±10 nm and proteoliposomes (D) to be 150±12 nm.

Liposomes and proteoliposomes from TE extract of chloroplast membranes were generated as described above. To check if the TE proteins were reconstituted into vesicles, the proteoliposomes were fractionated on a floatation sucrose gradient. The results obtained showed that ∼75% of the protein and PL was recovered at the 10–20% and 5–10% (w/v) sucrose interfaces. Proteoliposomes with a low PPR settled at a low percentage of sucrose (Figure 3A). Collisional quenching of NBD probes with KI was performed to assess if the labeled PLs were symmetrically distributed across the membranes of liposomes and proteoliposomes. Analysis of the quenching data using the modified Stern-Volmer plot indicated that ∼53±1% and ∼58±3% of symmetrically labeled NBD-PC liposomes and proteoliposomes reconstituted by bio-bead assisted Triton X-100 removal method were accessible to iodide ions. Upon testing asymmetrically labeled liposomes generated by addition of NBD-PC after vesicle preparation indicated that majority (∼78±2%) of the NBD-PC's were accessible to iodide ions as expected (Figure 3B). To confirm the size of the reconstituted vesicles and to check if they are unilamellar after extrusion through a 100 nm membrane, DLS measurements of vesicles were performed and the effective diameter of liposomes was found to be 110±10 nm and that of proteoliposomes to be 150±12 nm (Figure 3C and D). The vesicles were also analyzed before extrusion and it was found to be ∼600 nm (data not shown). The SEM morphological analysis were in agreement with the DLS measurements and vesicles were found to be spherical and well-dispersed (Figure S2). Vesicle leakage was checked using calcein. Liposomes encapsulated with calcein did not show emission at 517 nm in presence or absence of dithionite but upon addition of 0.2% Triton X-100, the emission increased because the vesicles were rendered leaky (Figure S3).

### Biogenic membrane and ATP dependent flippase activity in proteoliposomes reconstituted with TE of chloroplast membranes

**Figure 4:**
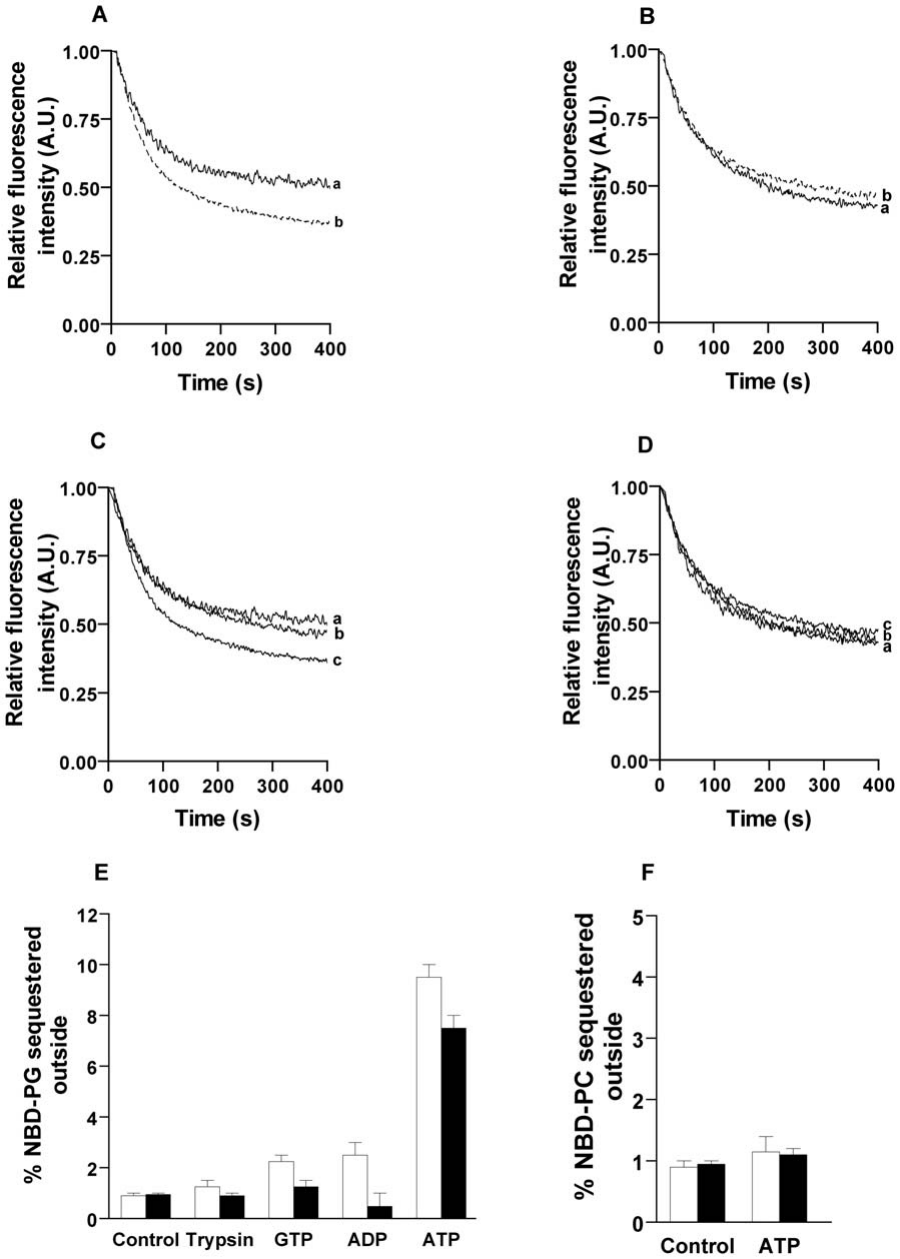
Localization of ATP independent and ATP dependent flippase activity in proteoliposomes reconstituted from TE of spinach chloroplast membranes. (A) The ATP independent translocation of NBD-PC in intact chloroplast (trace a – Liposome, b – 30 µl TE). (P<0.0001) (B) No energy independent translocation of NBD-PG in proteoliposomes from intact chloroplast (trace b) and liposomes (trace a) (p<0.05) (C) Energy independent translocation of NBD-PC (trace c) and no energy independent translocation of NBD-PG (trace b) in proteoliposomes reconstituted with envelope membranes and liposomes (trace a) (p<0.001) (D) No energy independent translocation of either NBD-PC or NBD-PG in proteoliposomes reconstituted with thylakoids (p<0.01). (E) Proteoliposomes were treated with 20 mM dithionite to make inside labeled vesicles and NBD-PG flippase activity from in to out was monitored in an energy dependent manner. The bar diagram represents proteoliposomes reconstituted with envelope membrane (white) and thylakoids (black) quenched with dithionite (control), NBD-PG proteoliposomes incubated with 5 mM GTP, ADP, ATP and 10 mg/ml trypsin quenched with dithionite (p<0.05) (F) Inside labeled vesicles of envelope membrane and thylakoid reconstituted proteoliposomes were prepared and NBD-PC flippase activity from in to out was monitored in an ATP dependent manner. The bar diagram represents proteoliposomes reconstituted with envelope membrane (white) and thylakoids (black) quenched with dithionite (control), NBD-PC proteoliposomes incubated with 5 mM ATP and quenched with dithionite (p<0.05). All the experiments were carried out 2 independent times.

Liposome showed only 52±3% quenching and the data was fitted to single exponential decay kinetics (Figure 4A–D) [9]–[15]. Proteoliposomes generated by reconstituting TE of intact chloroplast membrane proteins from spinach shows biphasic kinetics and the percentage of dithionite quenching was 60±2% (Figure 4A, R ^2^ = 0.98, p<0.0001). The data was fitted to double exponential decay kinetics as described in other published reports [9]–[15]. The results suggested that the proteoliposomes generated from TE of intact chloroplast was capable of translocating PC in an ATP independent manner (Figure 4A). In order to check whether TE of intact chloroplast membrane can also flip other PLs, we reconstituted NBD labeled PG as it is present in chloroplast membrane into proteoliposomes and checked for flippase activity. Interestingly, it has been found that the flippases in chloroplast was not able to translocate PG (Figure 4B, R ^2^ = 0.99, p<0.05). Reconstitution of TE derived from envelope membrane with NBD-PG and NBD-PC showed flipping of only PC (60±2%) in an ATP independent manner (Figure 4C, R ^2^ = 0.98, p<0.001). Similarly, TE extract from thylakoids were reconstituted and ATP independent activity of neither PC nor PG was observed and data fitted to single exponential kinetics (Figure 4D, R ^2^ = 0.98, p<0.01). To check for ATP dependent activity, we generated proteoliposomes and treated it with 20 mM dithionite to form inside labeled vesicles which were incubated with 5 mM ATP as described earlier [37] and assayed for flippase activity. Interestingly, it has been found that envelope membranes and thylakoids have ATP dependent flippase activity which involves translocation of NBD-PG from inside to outside. Also negligible activity was found upon treatment with GTP and ADP (Figure 4E, White bar represents envelope membranes and black bar represents thylakoids). This confirmed NBD-PG translocation was only ATP dependent. On treatment with trypsin (protease inhibitor), activity was not observed confirming it to be protein mediated (Figure 4E). ATP dependent NBD-PC translocation was not observed in both envelope membrane and thylakoid preparations (Figure 4F). These results suggest that envelop membranes have ATP independent PC and ATP dependent PG translocation activity; whereas thylakoid membranes have only ATP dependent PG activity. To further characterize the ATP independent biogenic membrane flippase activity, we used intact chloroplast membranes as the source.

### Kinetics of biogenic membrane flippase activity in chloroplast membrane

**Figure 5:**
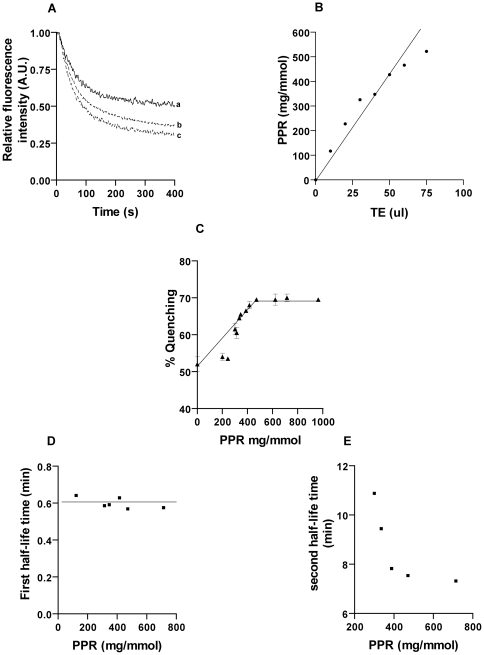
Biogenic membrane flippase activity in intact chloroplasts. (A) The level of translocation of NBD-PC increases with an increase in the amount of protein in the proteoliposome reconstituted with intact chloroplast (trace a – Liposome, b – 30 µl TE, c- 60 µl TE) (P<0.001) (B) Linear relationship between the amounts of TE to PPR in the reconstituted vesicles. (C) The extent of dithionite reduction of NBD-PC depends on protein to PL ratio (PPR). Flippase activity increases proportionately with an increase in PPR and saturates at a PPR∼400 mg/mmol. Above this value the percentage of fluorescence quenching remains unchanged for proteoliposomes. ***Kinetics of NBD-PC flipping*** - The calculation of half-life time was performed using the equation F(t) = F_0_−[A_1_ exp(−k_1_t)+A_2_ exp(−k_2_t)] where, F(t) is the fluorescence as a function of time and F_0_ is the fluorescence intensity at time = 0 s (i.e. initial fluorescence of the vesicles), k_1_ and k_2_ are the rate constants for the first (fast) and second (slow) phases respectively. A_1_ and A_2_ are the amplitudes of the fast and slow phases respectively. (D) The first phase half-life time remained constant at ∼0.6 min±0.05 min which represents the quenching of fluorescently labeled outer leaflet lipids. (E) The second phase half-life time decreased with increase in PPR suggesting that the kinetics of flipping increased and stabilized at a PPR of ∼400 mg/mmol. All the experiments were carried out 3 independent times.

Reconstituted liposomes showed only 52±3% quenching and the kinetic data were fitted to single exponential decay (R^2^ = 0.98, p<0.0001) with half life of ∼0.6±0.05 min. Proteoliposomes generated by reconstituting TE of chloroplast membrane proteins from spinach shows biphasic kinetics (R^2^ = 0.99, p<0.0001) and the percentage of dithionite quenching was 60±2% and upon increasing the concentration of TE used for reconstitution quenching increased to 65±1% (Figure 5A). This implies that second phase of quenching is slower because of transbilayer movement of PLs from inner leaflet to the outer leaflet. To study the effect of amount of TE addition during reconstitution on flippase activity, we generated proteoliposomes with varying amounts of protein to PL ratio (PPR) and assayed for flippase activity (Figure 5B). We found that with increase in TE addition during reconstitution, the amount of NBD quenching increases and get saturated at ∼400 mg/mmol. Further increase in PPR did not show any improvement in % quenching (Figure 5C). The first and second half-life time for all the experiments were calculated using the double exponential decay fit. Irrespective of the protein content in reconstituted vesicles, it was found that the first half-life time remained constant at 0.6±0.05 min (Figure 5D). The second half life time of 10±1 min was constant till the PPR was 400 mg/mmol. Beyond this level, the second half-life time decreased with increase in PPR (Figure 5E). This clearly suggested that beyond PPR of 400 mg/mmol, the % NBD quenching did not change but the kinetics of flipping gets altered. At higher PPR, second half-life time decreased, implying that kinetics of PC flipping became faster [12]–[13].

### Protease treatment

**Figure 6:**
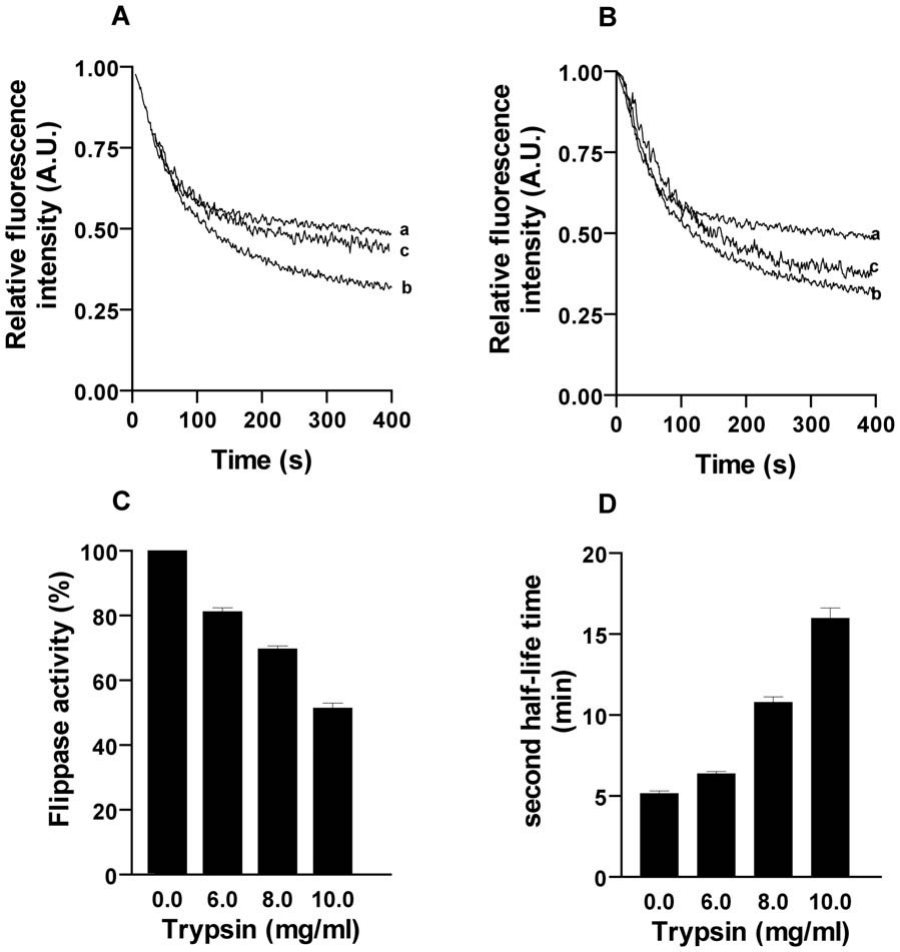
Effect of protease (trypsin) on flippase activity from spinach chloroplast membrane proteins. (A) Trypsin treatment before reconstitution – TE was treated with 10 mg/ml trypsin and reconstituted (trace c), trace a and b represent liposome and untreated proteoliposome (control), (p<0.001) (B) Trypsin treatment after reconstitution – Proteoliposomes were incubated with trypsin and incubated at 37°C for 30 min and flippase assay was carried out, trace a and b represent liposome and untreated proteoliposomes, trace c shows proteoliposomes treated with trypsin, (p<0.01)(C) Bar diagram shows the decrease in flippase activity when proteoliposomes were treated with varying concentrations of trypsin, (p<0.01) (D) The kinetics of flipping was altered with increase in trypsin concentration.

To further confirm that it is purely a protein mediated translocation, we treated the TE with trypsin (10 mg/ml final concentration) prior to reconstitution and assayed for flippase activity after reconstitution. It was found that vesicles generated from the trypsin treated TE showed 52±3% activity similar to liposomes (Figure 6A) and the kinetics also fitted to single exponential decay fit (R^2^ = 0.97, p<0.001). This suggests that flippase activity was completely inhibited when TE was treated with trypsin and confirmed that PC flipping is protein mediated. To have more insight into this, we also treated the proteoliposomes after reconstitution with varying concentrations of trypsin. The treated samples were then assayed for their ability to translocate NBD-PC. Flippase activity decreased in dose dependent manner and gets saturated with 10 mg/ml trypsin treatment (Figure 6B and C). Trypsin treatment after reconstitution could not completely inhibit flippase activity suggesting that some active sites of flippase were buried inside the membrane bilayer during reconstitution. The kinetics was fitted to a double exponential decay equation. The first half-life time was unaltered (0.6±0.05) whereas the second half-life time increased with increase in trypsin concentration implying that initial trypsin concentration was too less to access and modify the active flippases but saturated at 10 mg/ml trypsin and the kinetics of PC flipping was also affected by trypsin treatment (Figure 6D).

### Effect of protein modifying reagents on biogenic membrane flippase activity

**Figure 7:**
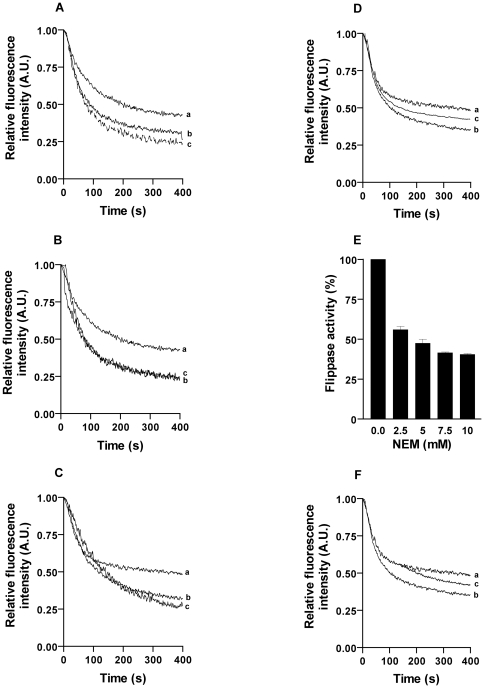
Effect of protein modifying reagents on the flippase activity. Proteoliposomes and TE were incubated with desired concentration of protein modifying reagents at 37°C for 30 min. (A) Effect of DEPC on flippase activity (trace a- liposome, b- proteoliposome, c- proteoliposome treated with 20 mM DEPC) (B) Effect of AEBSF on flippase activity (trace a- liposome, b – proteoliposome, c- proteoliposome treated with 5 mM AEBSF) (C) Effect of PG on flippase activity ( trace a- liposome, b-proteoliposome, c-proteoliposome treated with 5 mM PG. (p<0.001) ***Effect of NEM on flippase activity.*** (D) Effect of NEM treatment on proteoliposomes (trace a- liposome, b- proteoliposomes, c- vesicle treated with 40 mM NEM). (E) Effect of dose dependent NEM treatment on proteoliposomes. (F) Effect of NEM treatment on TE extract prior to reconstitution. (trace a- liposomes, b- proteoliposomes, c- TE treated with 40 mM NEM) (p<0.01).

Protein modifying reagents have been used to understand the critical amino acids responsible for flippase activity and whether more than one pool of flippases are present in biogenic membranes [12], [25], [40], [41]. In this study we checked sensitivity of flipping of NBD-PC to chemical modifications with various protein modifying reagents including N-ethylmaleimide (NEM) for cysteine, diethyl pyrocarbonate (DEPC) for histidine, 4-(2-aminoethyl)benzenesulfonyl fluoride hydrochloride (AEBSF) for serine and phenyl glyoxal (PG) for arginine [42]–[45]. Protein modifiers were used at varying concentrations in a dose dependent manner. TE prior to reconstitution and proteoliposomes (∼400 mg/mmol) were treated and incubated for 30 min at 37°C and assayed for flippase activity. TE treated with modifiers showed that PC flipping was affected only upon NEM treatment (Figure 7). Inhibition of flippase activity was calculated to be 60±2%. The other modifiers did not show any decrease or loss of activity beyond the concentrations used (20 mM of DEPC and 5 mM of PG and 5 mM of AEBSF) (Figure 7A, B and C). Thereafter proteoliposomes also were treated with varying concentration of modifiers and NEM showed a dose dependent inhibition of flippase activity (Figure 7D and E).

### Scramblase activity

**Figure 8:**
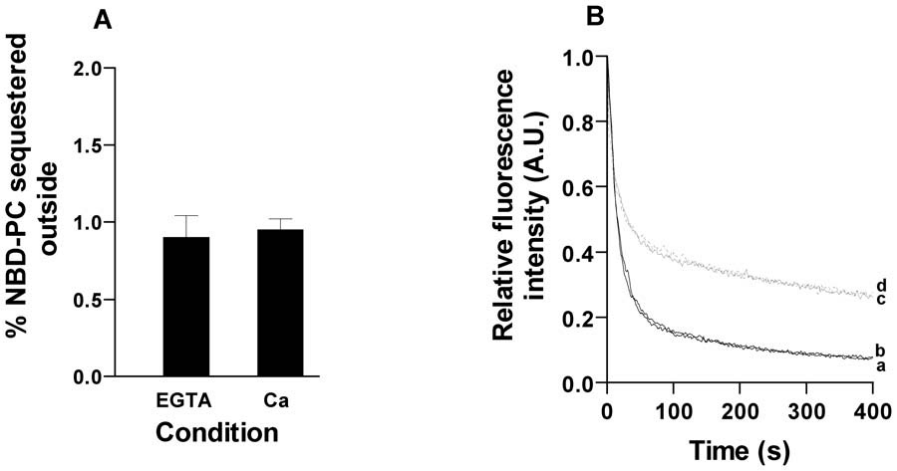
Scramblase assay for inside labeled and outside labeled proteoliposomes. (A) Inside labeled scramblase assay, both EGTA (control) treated (4 mM) and Ca^2+^ treated (2 mM) proteoliposomes showed same level of quenching. (B) Outside labeled scramblase assay, both showed same level of quenching confirming absence of scramblase activity (trace a – EGTA treated vesicles at t = 0 h, trace b - Ca^2+^ treated at t = 0, trace c- EGTA treated vesicles at t = 3 h, trace d – Ca^2+^ treated vesicles at t = 3 h).

To check if the flippase activity present in chloroplast membranes is calcium mediated phenomenon like scramblase, we performed an assay to confirm the same. Scramblase activity in vesicles containing chloroplast membrane (PPR∼400 mg/mmol) was performed using both inside labeled and outside labeled NBD-PC vesicles. Figure 8A and B shows that there was no calcium dependent increase in the level of quenching of NBD-PC reconstituted proteoliposomes. These results confirmed that the protein mediated flippase activity was not due to scramblase but due to biogenic membrane flippase which is consistent with the earlier reports that scramblase is localized only in the PM.

## Discussion

The synthesis of new membrane lipids majorly occurs in sub-cellular compartment of eukaryotes from where they are sorted to different organelles of the cell. Plant cells contain an additional sub-cellular compartment, the chloroplast not present in animal cells which comprises the extensive photosynthetic membrane system. Due to the heterogeneity of the chloroplast they cannot rely only on PLs as their membrane component. The plastid membrane is made up of a wide variety of lipids which serve numerous biochemical functions and also allow plants to adapt to fluctuating environmental conditions such as phosphate deprivation [46]. They are largely made up of glycolipids which include galactolipids and sulpholipids. The lipid composition and function of the outer and inner membrane is distinct. The outer and inner envelopes are involved in de novo synthesis of lipids, mainly fatty acids whereas the thylakoids lack lipid synthesizing machinery [47]. Fatty acids being the building blocks of biological membranes have to be exported from the plastid to the ER where they are assembled into glycerolipids which is then transferred to extraplastidic membranes under phosphate limiting growth conditions [48]–[50]. The exact mechanism for lipid trafficking is not completely understood though possible modes are through vesicular transport, membrane contact sites or by means of specific lipid transporters.

A recent report showed the first evidence of biogenic membrane flippase activity in ER isolated from plants [25]. Chloroplast being the other main lipid synthesizing organelle in plants, we hypothesized the presence of biogenic membrane flippase activity in chloroplast. To prove our hypothesis we isolated intact chloroplast membranes from spinach leaves using multiple percoll gradient protocol as used in proteome analysis and confirmed the purity of isolated fractions by specific marker enzymes as described in materials and methods. Intact chloroplasts were later fractionated to obtain envelope membranes and thylakoids. Liposomes and proteoliposomes were prepared as described previously by detergent removal using SM-2 Biobeads. A low bead/detergent ratio was maintained to promote the micellar to lamellar transition for the first 3 h of reconstitution as described in earlier reports, and later the Biobead concentration was increased to accelerate removal of detergent (Triton X-100) that might interact in subsequent flippase activity measurements. It has been shown that vesicles prepared in this manner are unilamellar with a mean diameter of 150 nm after extrusion and the key factor in determining the rate of detergent removal was the availability of free bead surface [51]. Triton X-100 absorbance at 275 nm was used to monitor the removal of detergent during the reconstitution [14]. The vesicles formed were unilamellar after extrusion through a 0.1 µm filter ∼11 times and of uniform size as observed by DLS analysis as the polydispersity index observed was low (0.13±0.03). Before proceeding with the flippase assay, we checked whether the NBD probes were incorporated symmetrically into the liposomal and proteoliposomal membranes by collisional quenching using KI. The results indicated that vesicles contained equivalent amount of NBD fluorophores in each leaflet, ∼46% NBD probes were found to be inaccessible to iodide quenching whereas, asymmetrically labeled liposomes showed ∼75% accessibility confirming that the NBD-PLs are symmetrically distributed across the membranes of the reconstituted vesicles and that the distribution is largely unaffected by the presence of membrane proteins. The detergent extract of chloroplast membranes including envelope and thylakoids were reconstituted *in vitro* into proteoliposomes and assayed for flippase activity. The flippase activity was measured by quenching of fluorescently labeled PLs by sodium dithionite, a membrane impermeable reagent [25], [52]. When treated with dithionite, liposomes showed 52±3% quenching of outer leaflet lipids and detergent extract of intact chloroplast reconstituted proteoliposomes showed 65±1% quenching which fits to two phase kinetics (R^2^ = 0.99, p<0.0001). The first half-life was around 0.6±0.05 min and half-life time of the second phase was 7±0.03 min. Both the first and second half-life is slightly higher than that reported in plant ER and other biogenic membrane flippases. Reconstitution with increasing concentration of TE showed proportional increase in activity establishing the relation between increasing protein to PL ratio with flippase activity. Sucrose floatation gradient was performed to reconfirm that TE proteins were reconstituted into vesicles. The percentage of NBD-PC quenched increased linearly to a PPR of ∼400 mg/mmol and attained saturation. The saturation value is interpreted as the proteoliposomes containing at least one functional flippase [11]–[13]. Also, beyond the PPR ratio of ∼400 mg/mmol, the second half time reduced with increase in rate of flipping. However, the PPR saturation value obtained in this study was almost 10 times of that obtained for spinach ER (40 mg/mmol). Compared to plants, saturation value is very low in rat liver microsomes (12 mg/mmol), yeast (10 mg/mmol) and *Bacillus subtilis* (40 mg/mmol) [12], [14], [25], [52]. This suggests that the abundance of flippase in plant biogenic membranes is usually lower compared to that found in other eukaryotes [11].

Since the function of biogenic membrane flippases reported earlier were not specific to PL head group in plants and animals [13], [16], [25], we reconstituted the intact chloroplast TE extract with NBD-PG and NBD-PE. Surprisingly, both NBD-PG and NBD-PE reconstituted proteoliposomes did not show any biogenic membrane flippase activity suggesting that it could be a novel pool of energy independent flippase specific to PC and different from that found in plant ER. Chloroplast membranes are mainly constituted of glycolipids (galactolipids and sulpholipids) and very small amounts of PLs. The PLs present in the outer envelope include 32 mol% of PC and 10 mol% PG of total lipids, inner envelope membranes have 9 mol% PG and thylakoids have 7 mol% PG respectively [53]. Since PG is present in considerable amount in the envelope membranes and thylakoids, and did not flip in an energy independent manner we explored the possibility of ATP dependent translocators. We observed that inside labeling of PG in both envelope membranes and thylakoids showed flippase activity in an energy dependent manner from inside to outside. It was confirmed that the translocation requires energy derived from ATP hydrolysis because negligible activity was found when GTP and ADP were used as the nucleotide substrates (Fig. 4E). Inside labeling of PC (Fig. 4F) and PE (data not shown) did not show ATP dependent activity. This is consistent with the plastid membranes as they lack PE and PC flipping (present only in outer envelope membrane) was observed in an ATP independent manner.

Flippase activity being rapid and bidirectional we wanted to confirm that the ATP independent and ATP dependent activity obtained was protein mediated. We used trypsin a protease inhibitor prior to reconstitution and after reconstitution and incubated the samples for 30 min at 37°C. Flippase assay was done as mentioned previously and the results clearly show that protease treatment resulted in decrease of NBD quenching implying that trypsin treatment resulted in reduction of flippase active vesicles. Dose-dependent treatment of proteoliposomes showed proportionate decrease in activity ∼47% at 10 mg/ml trypsin concentration with increase in half-life time, while in plant ER (75±2% for NBD-PC and 82±4% for NBD-PE flipping) the decrease was much more significant [25]. Also we treated the TE with protease just to ensure that all flippase active sites are exposed and it showed ∼90±5% decrease in activity. This suggested that treatment after reconstitution could affect only ∼50% activity because only half the protein is digested as it was rightly oriented (inside-out or right-side-out).

Since protease treatment confirmed the activity to be protein mediated we checked the sensitivity of flipping in both NBD-PC and NBD-PG to chemical modifications. Protein modification studies have been reported in a variety of membrane localized transporters to decipher which amino acids are critical for functionality of a protein. Cysteine is a critical amino acid present in the class of flippases as seen from earlier reports [12], [13]. We used the common modifying reagents which included DEPC (His modifier), NEM (Cys modifier), AEBSF (Ser modifier) and PG (Arg modifier). Interestingly NBD-PC flippase activity was not altered when either the proteoliposomes or TE were treated with DEPC, AEBSF and PG. But treatment of vesicles with NEM showed ∼60% loss of activity (Figure 7E). The inhibition of flippase activity by NEM was comparable (60±2%) before (TE treatment) and after (vesicle treatment) reconstitution. Whereas ATP dependent NBD-PG flippase activity was not altered by any of the modifiers stated above. The effect of modifiers was tested systematically to ensure that the knock-out of functional flippases was not due to insufficient reagent. Earlier experiments with plant ER showed significant change in activity when treated with modifiers and rat liver flippase showed that NEM an analogue of DTNB reduced flippase activity by ∼40% and had similar effect on NBD-PC and NBD-PE [13] whereas, in plant ER inhibition of PE and PC were not identical. NBD-PC flipping was affected by ∼60% after DEPC and DTNB treatment, ∼50% after PG and ∼10% after AEBSF treatment respectively [25]. In this study NBD-PC flipping was not sensitive to DEPC, AEBSF and PG suggesting the flippases present in plant ER and chloroplasts belong to distinct class (Figure 7). Flipping activity of ATP dependent NBD-PG was also studied using the above mentioned modifiers but no inhibition of flippase activity was observed suggesting strongly the presence of more than one pool of flippases in chloroplast.

To rule out the possibility that activity obtained might be because of a pool of scramblases which is also involved in rapid, bi-directional scrambling of PLs in a Ca^2+^ dependent manner we assayed for scramblase activity using both inside labeled and outside labeled vesicles with no activity (Fig. 8). This confirmed that the protein dependent activity was not scramblase mediated and was only due to the presence of biogenic membrane flippase which is consistent with reports that scramblase is localized in PM.

### Conclusions

In summary, we show for the first time evidence of biogenic membrane flippase activity in proteoliposomes reconstituted from TE of spinach chloroplast envelope membranes. But unlike the usual biogenic membrane flippase which does not distinguish between PL head-group, we observed chloroplast membranes could translocate only NBD-PC. We also showed evidence for ATP-dependent PG translocation in envelope membranes and thylakoids suggesting the presence of more than one pool of flippases in chloroplast membranes. The biogenic flippase activity (PC) was confirmed to be protein mediated by trypsin treatment before and after reconstitution. Saturation of flippase activity at ∼400 mg/mmol suggests that the abundance of spinach chloroplast flippase is very low compared to spinach ER. Also, spinach ER flippase exhibited inhibition of activity with DEPC, DTNB, PG and AEBSF in varying degrees while spinach chloroplast flippase was inhibited exclusively by cysteine modifier (NEM) affecting the kinetics of flipping. These results strongly suggest that the lipid translocators in plant ER and chloroplast are distinct. However there might be other population of plastid flippases specific for galactolipids which could not be studied because fluorescent analogs of galactolipids are not commercially available.

## Supporting Information

Figure S1 **Schematic representation of ATP dependent flippase assay.** (A) Energy dependent activity – Inside labeled vesicles are prepared by treating the symmetrically labeled vesicles with 20 mM dithionite which are then incubated with ATP/GTP/ADP and control is incubated without energy source. (B) Inside labeled vesicles are incubated with and without (control) energy source and assayed for activity by addition of dithionite.
(TIF)

Figure S2 **SEM pictures of proteoliposomes.** 10–20 µl of extruded vesicles (proteoliposomes) was placed on the surface of a glass coverslip which was then stuck on a SEM stub with a carbon membrane. The sample was coated with gold particles in a HITACHI E-1010 ion sputter and observed in a SEM (HITACHI S-4800). The mean size of the vesicle was estimated using the built in software. The vesicle size observed was found to be 120±30 nm.
(TIF)

Figure S3 **Vesicle leakage assay.** To determine leakage of vesicles calcein a water soluble dye was used as an internal marker as described earlier. Vesicles were prepared devoid of fluorescent lipids (A) and with NBD-PC labeled (B). The lipids were dried and solublized in reconstitution buffer containing 50 mM calcein, vesicles were prepared and leakage assay was performed as described in earlier reports by measuring fluorescence intensity at an excitation wavelength of 490 nm and emission wavelength of 517 nm. Traces a and b correspond to intensity in absence and presence of dithionite respectively. Trace c is the increase in fluorescence intensity due to calcein release upon addition of 0.2% Triton X-100 to the vesicles.
(TIF)
